# Proceedings of the Memphis Medical Society

**Published:** 1899-04

**Authors:** 


### PROCEEDINGS OF THE MEMPHIS MEDICAL SOCIETY.

*Stated Meeting February 21, 1899. Dr. B. F. Turner, President.*

Dr. E. C. Ellett presented a case of probable "Chancre of the Conjunctiva." The sore was situated at the inner end of the lid, and extended to both palpebral and ocular conjunctiva. It was of four days' duration, raised from the surface, with sharp edges, an indurated base, very little pain, and slight discharge. The preauricular and cervical glands on that side were enlarged. There was no history of any possible source of infection, except the use of a public towel. The patient was a young man of twenty-two, and had never had any previous venereal disease.*

Dr. Heber Jones thought that the location of the sore would so alter its appearance as to make a diagnosis very difficult. He agreed that it was at least suspicious, and advised waiting for the appearance of the secondary symptoms.

Dr. F. D. Smythe regards enlargement of the glands at the pos-

*NOTE.—Four weeks from the appearance of this sore the patient developed a macular syphilide.

terior border of the trapezius muscle, occurring in a clean person without tubercular antecedents, as pathognomonic of syphilis. The presence of this sign and the character of the lesion on the conjunctiva leads him to think that it is a chancre.

Dr. E. P. Sale spoke on "Occipito-Posterior Positions of Vertex Presentations." Next to L. O. A., R. O. P. is the most frequent position when the vertex presents. The labor is slow and painful, the patient nervous, the os poorly dilated, the uterus concave forward, and the chin may be felt above the pubis. The vaginal examination reveals the anterior fontanelle or forehead at the os. In the management of such a case the tendency is toward extension, and the keynote to treatment is flexion. The head engages its biparietal diameter between the promontory of the sacrum and the pubic spine, and is prevented from reaching the pelvic floor. The hand of the vectis may be introduced to supply sufficient resistance to induce flexion, or the head may be turned on one shoulder and thus released and allowed to descend. Version is sometimes resorted to, or a high forceps operation may be done. This is very difficult. If such a case is neglected, the occiput is likely to rotate into the hollow of the sacrum, which constitutes a difficult condition to deal with. If forceps are used the result is generally a ruptured perineum and an asphyxiated child; the child can usually be revived. When the patient is seen before labor and the position recognized, it may be converted into an L. O. A. by having the woman assume the knee-chest position for a few minutes two or three times a day, and then to lie on the right side.

Dr. Smythe usually is satisfied when he recognizes a head presentation, and does not investigate further. If delivery is not accomplished in a reasonable length of time, he applies the forceps.

Dr. Edwin Williams regards the forceps operation in occipito-posterior positions as very difficult.

The President related a recent case of occipito-posterior position in which the cervix was far back and high up. By hooking a finger in the cervix and drawing it forward flexion was produced, and rotation and delivery followed.

Dr. Sale said that the ordinary forceps might be applied reversed in these cases, or the straight forceps or vectis might be used. He thinks it well to explain at once to the patient the false position and probable slow labor.

Dr. Alfred Moore presented a symposium of "Therapeutic Notes" contributed by different members of the Society. Dr. Minor commended the action of a 5 per cent. solution of protargol in purulent conjunctivitis, and the A. C. E. mixture for general anesthesia. Dr. Turner contributed good results in pneumonia from the solid extract of veratrum viride, in quarter- and half-grain doses. Dr. Sale has found nosophen an excellent substitute for iodoform. Dr. Rice has had good results from the inhalation of ethyl iodide in chronic cough and chronic inflammation of the respiratory tract. Dr. McKinney uses cocain, nitrate of silver, camphor, menthol and chromic acid more than any other remedies. Dr. Williams has found argentamin of value as an injection in gonorrhea. It is a phosphate of nitrate of silver, and contains 10 per cent. of nitrate of silver. It does not precipitate with sodium chloride or albumin. It is antiseptic, and penetrates the mucous membrane. It is quite irritating, and it is to be used $\frac{1}{4000}$ in the anterior urethra and $\frac{1}{5000}$ in the posterior urethra. In a case of acute gonorrhea injections every four hours produced a cure in sixty hours. Dr. Smythe finds pilocarpin the diaphoretic and expectorant *par excellence*. Dr. Ellett mentioned the action of the suprarenal extract, in the proportion of 1 to 10 of cold saturated boric acid solution, as a powerful vasomotor astringent. Mucous membranes are blanched under its influence, and may be bloodlessly cut. Argonin has given excellent results in gonorrheal ophthalmia of infants and adults, and in purulent ophthalmia from other causes. It is used in 5 per cent. solution every three hours. Holocain and orthoform have been made the subject of favorable reports on former occasions.

Dr. Richmond McKinney has had good results from the use of the suprarenal extract locally in acute laryngitis. Orthoform has yielded very unsatisfactory results in his hands.

Dr. Smythe has had excellent results from argonin in 5 per cent. solution in two cases of ophthalmia neonatorum.

Dr. E. E. Haynes had, in a bad case of ophthalmia neonatorum, used an 8 per cent. solution of argonin in one eye, and nitrate of silver in the other. The former acted so much the better that it was subsequently used in both. The recovery was perfect in the eye treated with argonin, but the one in which the silver nitrate was used shows a scar from corneal ulceration.

Dr. Ellett spoke of favorable results obtained in trachoma by the use of cupric cataphoresis. The principles involved were explained. The eye is cocainized, and a pure copper electrode, attached to the positive pole, applied to the everted lid. A current of two milliamperes is as strong as can be tolerated. The electrode for the application was shown. The treatment is less severe than the application of sulphate of copper.

Dr. Sale thought that antisepsis should prevent the occurrence of ophthalmia neonatorum, and considered its appearance as somewhat of a reproach to the obstetrician, though he has seen it developed after due cleanliness had been observed. He thinks the Credé method for preventing this disease barbarous.

Dr. Ellett said that the Credé method was severe but effective. In Leipzig the percentage of cases was reduced from 10.8 per cent. to 0.2 per cent., and in the Memphis City Hospital Dr. Ellett has seen very striking results. The disease has been seen to develop, however, in spite of this treatment, and has, in a few cases, been contracted *in utero*.

The President corroborated Dr. Ellett's statements as to infection occurring *in utero*.

---

### INFANTILE URETHRITIS.

Professor Imerwot-Jassy, in the *Archiv für Kinderheilkunde*, Bd. 22, Heft 5 u. 6, says that a large majority of the purulent urethritis met with in boys, which has been considered as catarrhal, is true gonorrhea. In ten cases between the ages of one and a half and eleven years gonococci, as well as the source of infection, were demonstrable. One case was complicated with epididymitis, and others became chronic in character. The treatment is the same as in adults (injections, etc.), but prophylactic measures are of the greatest value where members of the household, as the mother, sisters, or maids, are afflicted with leucorrhea.

